# Molecular biology of cantharidin in cancer cells

**DOI:** 10.1186/1749-8546-2-8

**Published:** 2007-07-04

**Authors:** Rolf Rauh, Stefan Kahl, Herbert Boechzelt, Rudolf Bauer, Bernd Kaina, Thomas Efferth

**Affiliations:** 1 State of Maryland Department of Health and Mental Hygiene, Maryland, USA; 2 Institute of Pharmaceutical Sciences, University of Graz, Graz, Austria; 3 Joanneum Research, Graz, Austria; 4 Institute of Toxicology, University of Mainz, Mainz, Germany; 5 Pharmaceutical Biology (C015), German Cancer Research Centre, Im Neuenheimer Feld 280, 69120 Heidelberg, Germany

## Abstract

Herbal medicine is one of the forms of traditional medical practice. Traditional Chinese medicine (TCM) and traditional Vietnamese medicine (TVM) are well-known for their long-standing tradition of herbal medicine.

Secreted by many species of blister beetle, most notably by the 'Spanish fly' (*Lytta vesicatoria*), cantharidin inhibits protein phosphatases 1 and 2A (PP1, PP2A). Blister beetle has been used in Asian traditional medicine to treat *Molluscum contagiosum *virus (MCV) infections and associated warts, and is now also used for cancer treatment. A combination of both genomic and postgenomic techniques was used in our studies to identify candidate genes affecting sensitivity or resistance to cantharidin. Cantharidin was not found to be related to multidrug resistance phenotype, suggesting its potential usefulness for the treatment of refractory tumors. Oxidative stress response genes diminish the activity of cantharidin by inducing DNA strand breaks which may be subject to base excision repair and induce apoptosis in a p53- and Bcl2-dependent manner.

Cantharidin is one of many natural products used in traditional Chinese medicine and traditional Vietnamese medicine for cancer treatment. Combined methods of pharmaceutical biology and molecular biology can help elucidate modes of action of these natural products.

## Background

Herbal medicine represents a traditional form of medical practice in human history. Current ethnobotany and ethnopharmacology focus on the systematic exploration of medicinal herbs among folk medicines [1].

Half a century after the launch of chemotherapy for tumor treatment [2], anti-neoplastic drugs is now indispensable for treating hematopoietic malignancies. The concept of combination therapy in oncology is based on the notion that leukemia cells resistant to one drug may be susceptible to other drugs. Clinically, the success of combination treatments can be hampered by the development of broad-spectrum or multidrug resistance (MDR). Two strategies to cope with this problem are to modulate multidrug resistance by inhibitors of MDR-conferring proteins [3] or to develop new anticancer drugs without involvement in MDR phenotypes. Enormous efforts have been spent to develop such as resistance modulation to improve treatment for leukemia, but resistance modulators still frequently show intolerable high toxicity [4]. Natural products provide a rich source for developing novel drugs with anti-leukemia activities. The Natural Products Branch of the National Cancer Institute (USA) has collected and tested over 100,000 natural extracts of plants and invertebrates [5]. The vast experience of traditional medicines (e.g., TCM) may facilitate the identification of novel active substances. This approach has been successful. Camptothecin from *Camptotheca acuminata *represents only one outstanding example for such compounds derived from TCM [6]. Considering the severe limitations of current cancer chemotherapy, it would be desirable to have novel drugs which are active against otherwise resistant tumor cells.

In 1996, we started a research program on the molecular pharmacology and pharmacogenomics of the natural products derived from TCM [7]. This project became fruitful for the identification and characterization of compounds with anti-tumor and anti-viral activities. Apart from artemisinin and its prominent semi-synthetic derivative artesunate which are both approved drugs [8,9], we have analyzed cellular and molecular mechanisms of several other chemically characterized natural products derived from TCM, e.g., arsenic trioxide, ascaridol, berberine, cantharidin, cephalotaxine, curcumin, homoharringtonine, luteolin, isoscopoletin, scopoletin and others [10-30]. Furthermore, several novel bioactive compounds, namely tetracentronsine A, tetracentronside A, B and C, the two novel α-tetralone derivatives, berchemiaside A and B, and the novel flavonoid quercetin-3-*O*-(2-acetyl-α-L-arabinofuranosid), were described and analyzed in our investigations, [31-33]. Artemisinin displays a marked anti-malarial activity [34]. Various derivatives (artesunate, artemether, arteether, artelinate) have been synthesized to improve this anti-malarial activity.

In 2002, we began a study on the antiviral effects of artesunate. We demonstrated for the first time that artesunate inhibits NF-κB activity, leading to the inhibition of viral replication. NF-κB is involved in the transcriptional regulation of early and late proteins of human cytomegalovirus (HCMV) necessary for viral replication [35]. Artesunate also acts against cytomegaloviruses *in vivo *[36]. We also showed that the antiviral activity of artesunate is not limited to HCMV. Herpes simplex virus 1, hepatitis B and C viruses and others can also be inhibited by artemisinin and artesunate [35,37,38].

TCM is well-known for its unique diagnosis and treatment system, whereas other traditional medicines in Asia, such as TVM, have not gained the same acceptance in medical practice. Profoundly influenced by TCM, TVM exhibits its uniqueness via an influence from indigenous medicine of southern Vietnam. Detailed surveys of TVM and TCM can be found in recent publications [39-41].

## Cantharidin

### Screening of extracts

A major problem in conventional cancer chemotherapy is the severe side-effects on normal tissues prevent treatment with doses sufficient to kill all cancer cells, which in turn fosters the development of drug resistance. There is an urgent need to develop new drugs with improved resistance modulation for tumor therapy. It is the current situation in cancer chemotherapy in Western medicine that prompted us to study TCM and TVM. We have been searching for natural products from medicinal plants with activity against cancer cells.

**Figure 1:**
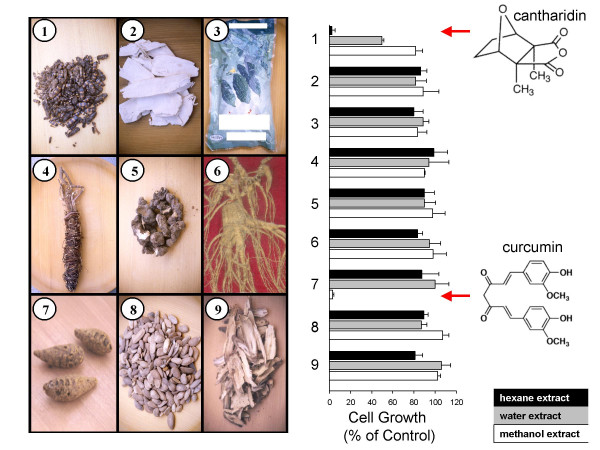
Medicinal plants and animals used in Vietnamese medicine and cytotoxicity of hexane, water or methanol extracts (10 μg/ml) to CCRF-CEM leukemia cells. (1) *Lytta vesicatoria *(whole beetles), (2) *Pueralia lobata *(roots), (3) *Momordica charantia *(roots, branches), (4) *Momordica charantia *(fruits), (5) *Dichroa febrifuga *(roots), (6) *Panax ginseng *C.A.Mey., (7) *Curcuma longa *(rhizoma), (8) *Trichosanthes kirilowii *Maxim (kernels), (9) *Glycyrrhiza uralensis *(roots). Growth inhibitory activity was measured using a growth inhibition assay [16].

In this study, we focused on medicinal plants and animals used in Vietnam. They were bought by of the authors at medicinal markets in Ho Chi Minh City, Vietnam (Figure 1). The criteria for medicinal plants and animals to be included in this study were their traditional use to treat cancer. The material was further processed by extraction with solvents of different polarity (petroleum ether, n-hexane, methanol, chloroform, ethyl acetate, or water). Extracts with organic solvents were made in a Soxhlet apparatus, while water extracts were made as decoctions imitating the original medicinal use. The aim of this approach was to divide plant constituents into fractions of different polarity upon extraction.

Only two out of the 27 extracts, namely *Curcuma longa *and blister beetle ('Spanish fly', *Lytta vesicatoria*), reduced cell growth of human CCRF-CEM leukemia cells to below 20% of untreated controls (Figure 1). We further tested curcumin and cantharidin, the active ingredients of *Curcuma longa *and of *Lytta vesicatoria*, respectively [16].

### Multidrug resistance

Parental CCRF-CEM leukemia cells and their multidrug-resistant sub-lines CEM/ADR5000, CEM/VLB100, and CEM/E1000 were used to investigate whether these two substances are able to inhibit tumor cell growth and to analyze the involvement of these compounds in multidrug resistance (MDR) phenotype. CEM/ADR5000 and CEMJ/VLB100 are characterized by over-expression of the MDR-mediating ATP-binding cassette (ABC) transporter P-glycoprotein (*MDR1, ABCB1*), while CEM/E1000 over-expresses the multidrug-resistance related protein 1 (*MRP1, ABCC1*) [42-44]. Cantharidin is more active than curcumin and no cross-resistance was observed in multidrug-resistant cell lines indicating that both drugs are not involved in the MDR phenotype (Table 1). Since cantharidin was active at lower concentrations compared to curcumin, we focused our efforts on cantharidin. An overview on the chemotherapeutic features of curcumin can be found in a recent publication [45].

**Table 1:** Fifty percent inhibition concentrations (IC_50_) and relative resistance of curcumin and cantharidin in sensitive and multidrug-resistant CEM leukemia tumor cell lines [16]

	**Curcumin**	**Cantharidin**
**Growth inhibition assay:**		
CCRF-CEM	30 μM	20 μM
CEM/ADR5000	28 μM	17 μM
Relative resistance	0.9	0.9
**MTT-Assay:**		
CCRF-CEM	>13.5 μM	1.9 μM
CEM/VBL100	>13.5 μM	1.4 μM
Relative resistance:	n.d.	1.4
CEM/E1000	13.5 μM	0.76 μM
Relative resistance	n.d.	0.4

n.d.: not determined

### Use of canthardin in Asian and Western medicine

Topical application of cantharidin has a long tradition in Asian medicine for the treatment of warts caused by *Molluscum contagiosum virus *(MCV) infections [46], while the use of cantharidin to treat pediatric MCV infections in Western academic medicine has been found effective [47,48]. Blister beetles and cantharidin are also used in China and Vietnam to treat cancer [49]. Despite its usefulness, the potential poisonous effects of cantharidin would have fatal consequences in the event of careless mistakes in the use of this compound [50,51]. Not only is exact dosage is required for the use of cantharidin itself, but also for raw preparations of blister beetles to be used in traditional medicines. Cantharidin content varies among individual blister beetles as well as among different species. Blister beetles belong to the order *Coleoptera *which comprises approximately 1500 species.

In this review, our data are compared with well-known results regarding the molecular and cellular mechanisms of cantharidin in cancer cells.

## Pharmacogenomics of cantharidin

Cantharidin and norcantharidin (a demethylated cantharidin derivative, which also has clinical potential) are protein phosphatase 1 (PP1) and protein phosphatase 2A (PP2A) inhibitors [52-59]. This activity appears necessary for the growth inhibition activity of these compounds [57]. Protein phosphatases are involved, among others, in the regulation of multiple cellular processes including apoptosis, signal transduction pathways, cell cycle progression, glucose metabolism and calcium transport [60]. Thus, although the biochemical target of cantharidin and norcantharidin is known, the critical molecular pathways by which cantharidin and norcantharidin cause growth inhibition and cell death are unclear [61-63].

**Figure 2:**
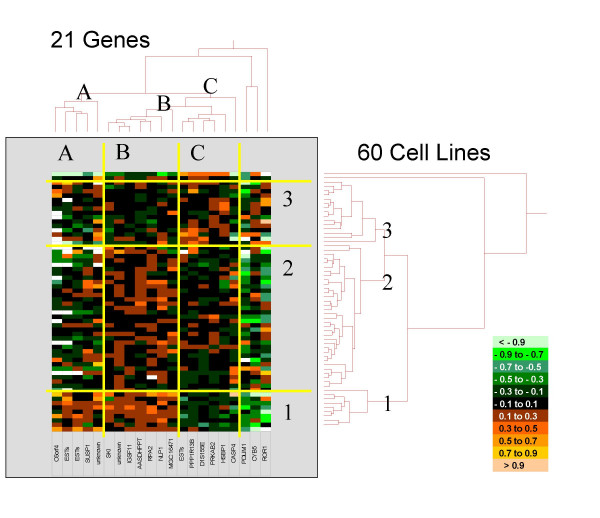
Dendrograms and cluster image map obtained by hierarchical cluster analysis (complete linkage method) of mRNA expression of 21 genes in 60 NCI cell lines. The genes are published [10]. The dendrogram on the right shows the clustering of cell lines and the dendrogram on the top shows clustering of genes. The cluster image map corresponds to each mRNA expression value obtained by microarray analysis. The expression values have been normalized and color-coded as indicated.  The figure is taken from [10].

In an attempt to identify the molecular determinants that predict sensitivity or resistance of tumor cells to cantharidin, we analyzed the microarray database of the National Cancer Institute (USA). Out of 9706 genes, 21 genes whose mRNA expression in 60 tumor cell lines correlated with the highest correlation coefficients to inhibition concentration 50% (IC_50_) values were selected by COMPARE analysis and false discovery rate calculation [10]. These genes were subjected to hierarchical cluster analysis to reveal whether the expression profiles of these genes are useful to predict sensitivity or resistance of cell lines to cantharidin. The mRNA expression of the 21 identified genes were subjected to hierarchical cluster analysis and cluster image mapping (Figure 2). The resulting dendrogram with the genes analyzed on the right can be divided into three major branches. The dendrogram on the top shows the cell lines and can also be separated into three major branches. By generation of a cluster image map from both dendrograms, areas with different mRNA expression levels became apparent (Figure 2). The distribution of sensitive or resistant cell lines on the dendrogram was significantly different indicating that cellular response to cantharidin is predictable by these genes [10].

While the specific functions of the proteins encoded by the 21 identified genes are diverse, it is intriguing that many of them are in one way or another involved in DNA damage response, DNA repair, and/or apoptosis [10]. Since cantharidin is an inhibitor of protein phosphatases 1 (PP1) and 2A (PP2A), PPP1R13B, the regulatory subunit 13B of PP1, is particularly interesting. PPP1R13B plays a central role in the regulation of apoptosis via its interaction with the tumor suppressor gene p53. It regulates p53 by enhancing DNA binding activity and transactivation function of p53 on the promoters of proapoptotic genes *in vivo *[64]. The role of PP1 in the repair of UV-induced DNA lesions [65] and the induction of cytosine arabinoside-induced apoptosis have been shown [66]. It is, therefore, reasonable to hypothesize that PPP1R13B also has specific functions in cantharidin-induced DNA repair and apoptosis. PP1 is one of the four major serine/threonine protein phosphatases, and new protein phosphatases are still emerging. They are crucial regulators of many cellular functions by altering the phosphorylation of target proteins. The level of phosphorylation is a well-controlled balance by the opposing actions of protein kinases and protein phosphatases. The PP1 holoenzyme consists of catalytic and regulatory subunits. Four catalytic (a, b, c, d) and more than a dozen regulatory subunits have so far been identified. The regulatory subunits modulate the substrate specificity and target the holoenzyme to specific subcellular localizations.

## Apoptosis and DNA damage and repair induced by cantharidin

The microarray analyses were used to find the genes responsible for the action of cantharidin. These studies showed that many apoptosis-related genes and genes involved in DNA damage and repair correlated with the IC_50 _values for cantharidin in the NCI cell line panel. We analyzed the relevance of apoptosis and DNA damage and repair induced by cantharidin in more detail. In a recent study, we reported that cantharidin induces apoptosis by a p53-dependent mechanism in leukemia cells [24]. Cantharidin causes both DNA single- and double-strand breaks. Colony forming assays with knockout and transfectant cell lines showed that DNA polymerase β conferred increased cell survival after cantharidin treatment, indicating that base excision repair rather than nucleotide excision repair is important for cantharidin-induced DNA lesions. Oxidative-stress resistant thymic lymphoma-derived WEHI7.2 variants are also more resistant to cantharidin. These data suggest that cantharidin treatment causes oxidative stress which damages DNA and triggers p53-dependent apoptosis.

It has been difficult to determine all critical events responsible for cantharidin-induced cytotoxicity. PP1 and PP2A which are inhibited by cantharidin [52-59], modulate a large number of cellular processes by counteracting the activity of kinases to provide the critical on/off switch for many pathways [60]. Cell cycle progression is one process where the increases and decreases of both kinases and phosphatases are necessary to complete the cycle. Studies from different laboratories agree that cantharidin and norcantharidin treatment results in a G_2_/M cell cycle block in many cell types [68-72]. However, several of these studies suggest that cell cycle blockade does not cause cantharidin-induced apoptosis [68-72].

Our data show that cantharidin treatment causes DNA strand breaks in CCRF-CEM cells [24]. DNA strand breaks have also been documented in oral cancer KB cells after norcantharidin treatment [73]. A correlation between an increase in the mRNA level for several DNA damage repair genes in the 60 cell line panel and resistance to cantharidin argues for DNA damage as a mechanistic component of cantharidin-induced apoptosis. This is consistent with the role of p53 in cantharidin-induced apoptosis in this study because one of the major functions of p53 is to induce apoptosis when DNA damage exceeds a threshold [74]. Moreover, p53 plays a role in norcantharidin-induced apoptosis in glioblastoma cells [68]. Phosphorylation of p53 stabilizes the protein [74,75]; inhibition of phosphatases may enhance the ability of p53 to exert its effect. The ability of Bcl-2 to protect against cantharidin-induced apoptosis seen in this study indicates that DNA damage-triggered mitochondrial pathway is involved. Mitochondrial dysfunction and activation of caspases involved in the intrinsic (mitochondrial) pathway of apoptosis have also been detected in other cell types after cantharidin or norcantharidin treatment [69,70,76-83]. A role of the Fas/CD95 extrinsic pathway of apoptosis has been reported [84], but not confirmed by other authors [85].

The cross-resistance of the oxidative stress resistant WEHI7.2 variants to cantharidin suggests that cantharidin causes oxidative stress which plays a role in cantharidin-induced apoptosis. Analogs of cantharidin increase xanthine oxidase activity which would increase intracellular reactive oxygen species (ROS) [86]. It is, therefore, tempting to speculate that oxidative stress is involved in the induction of DNA damage by cantharidin. Increase of endogenous ROS level has repeatedly been shown to cause significant DNA breakage [87].

Resistance to oxidative stress, increases of Bcl-2, or the presence of wild type p53 have a modest effect on cantharidin-induced toxicity. Mutational inactivation of PolB but not of ERCC1, key enzymes of base excision repair and nucleotide excision repair pathways respectively, exerted an effect on cantharidin cytotoxicity. This suggests that cantharidin induces non-bulky DNA lesions that are repaired by base excision repair but not by nucleotide excision repair. Lesions induced by oxidative stress are repaired by base excision repair and non-homologous end joining [88]. This suggests the possibility that multiple mechanisms are responsible for cantharidin-induced toxicity. In this study, for example, p53 status affected the IC_50 _of cantharidin, however, cells with mutated p53 still died. A p53-independent mechanism of cantharidin-induced cytotoxicity has been detected in hepatoma cells [70]. Because cantharidin and norcantharidin inhibit phosphatases, it would not be surprising that alterations in multiple pathways are critical for apoptosis. The microarray data comparing cantharidin treated and untreated HL-60 cells suggest that cantharidin affects multiple pathways [89]. Given that multiple mechanisms are involved in cantharidin-induced toxicity, the drug will likely be most effective against cancer cells with a specific phenotype. It is important to test whether this is a cancer cell that has acquired mutations in DNA repair pathways but retains wild-type p53 and sensitivity to oxidative stress.

## Discussion

Although the potential use of natural products is increasingly recognized in oncology, it has been estimated that so far only 5000 plant species have been properly studied for possible medical applications [90]. Considering that there are 250,000 to 300,000 plant species on this planet, the majority of this treasure still awaits retrieval.

The isolation of natural products and the elucidation of their chemical structures enable pharmacological and molecular biological investigations comparable to those conducted on chemically synthesized compounds to be conducted. The identification of target molecules relevant to diseases allows screening for natural products that are able to inhibit these targets. This may lay groundwork for the development of rational treatment of diseases such as cancer. This kind of research also opens avenues for the prediction of individual response of a cancer patient to therapy. We expect that strategies for individualized tumor therapies will lead to improved results for the patients. Small molecule inhibitors have the potential to increase tumor specificity and reduce adverse side effects on normal tissues. This concept of individualized tumor therapy is also of great importance for small molecule inhibitors derived from traditional herbal medicines such as TCM and TVM. Applying this strategy, we identified cantharidin as a potential drug candidate. These results are supported by findings that cantharidin does not cause myelosuppression in patients [70] and is effective against cells with a multidrug resistance phenotype [16,91], both of which are major obstacles of established anti-cancer drugs. A drawback of cantharidin is its acute toxicity due to its effect on mucus membranes and the urinary tract [49,73]. Understanding the mechanism of cantharidin action will help in the derivation of related compounds that have reduced toxicity while preserving the anti-tumor effect [92,93].

Cantharidin's ability to act against multidrug-resistant cells makes it an ideal compound for individualized cancer treatment. If multidrug resistance of a tumor can be detected by molecular and pharmacological means prior to standard chemotherapy, the therapy regimen may be altered and drugs that act against multidrug-resistant tumors (e.g., cantharidin) may be applied. Such escape treatment strategies are promising.

Our pharmacogenomic approach points to genes involved in oxidative stress response, DNA repair and apoptosis. The generation of hypotheses by genomic technologies and their verification by molecular biological methods provides an attractive approach. On the other hand, microarray technologies alone are not sufficient to elucidate molecular mechanisms of cytotoxic compounds in cancer cells. Microarray expression profiling can deliver candidate genes on a transcriptome-wide level. While this approach is much faster than traditional techniques, not all associations of genes with the response of tumor cells to a drug under investigation are of causal relationships and some may be even unrelated processes. Therefore, we should only take validated findings seriously.

## Conclusion

TCM and TVM are valuable sources for identifying potential natural products to treat cancer. In the case with cantharidin, the use of pharmacogenomic and molecular biological techniques help elucidate the modes of action of the natural product. While cantharidin is not involved in the multidrug resistance phenotype, it induces the generation of ROS and DNA damage, thereby leading to apoptosis.

## Competing interests

The author(s) declare that they have no competing interests.

## Authors' contributions

RR carried out the biological assays. HB coordinated the extraction of medicinal plants and animals and revised the manuscript. RB was the academic supervisor of SK. SK generated the extracts from medicinal plants and animals in RB's lab. BK coordinated the conduct of experiments of RR concerning apoptosis and DNA damage and repair. RR carried out the biological experiments in BK's lab. TE conceived the study, bought the medicinal plants and animals from Vietnam and drafted the manuscript. TE was the academic supervisor of RR. All authors read and approved the final manuscript.
